# Supplementary material for: The Bio-Diversity and the Role of Gut Microbiota in Postmenopausal Women with Luminal Breast Cancer Treated with Aromatase Inhibitors: An Observational Cohort Study
Source: Pathogens. 2022 Nov 26;11(12):1421. doi: 10.3390/pathogens11121421 (PMC9781910; doi:10.3390/pathogens11121421)
Supplement: Supplementary file 1 [file pathogens-11-01421-s001.zip › Figure S1.pdf]

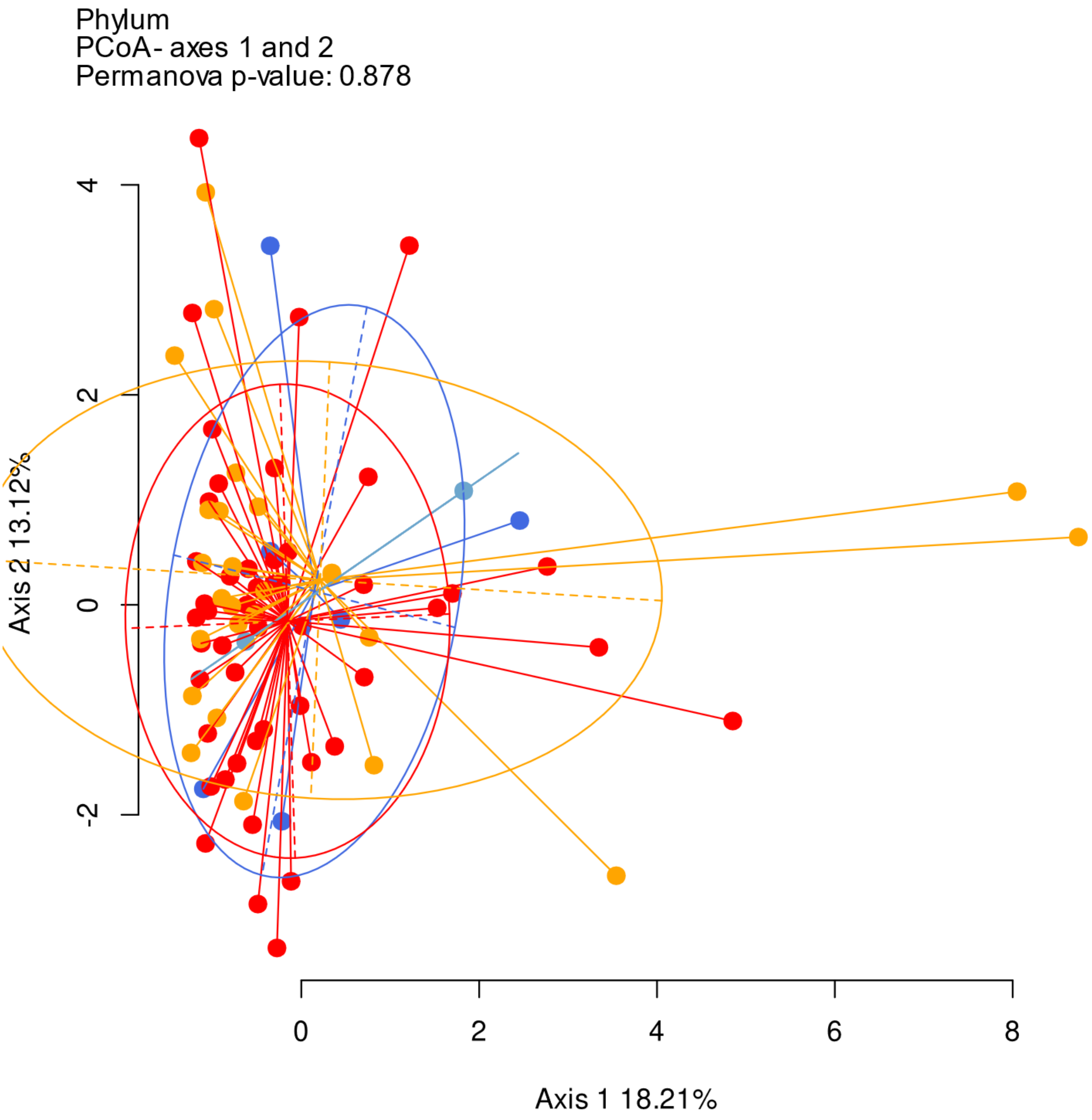
Phylum
PCoA- axes 1 and 2
Permanova p-value: 0.878
Axis 2 13.12%
4
2
0
-2
0
2
4
6
8
Axis 1 18.21%

(a)

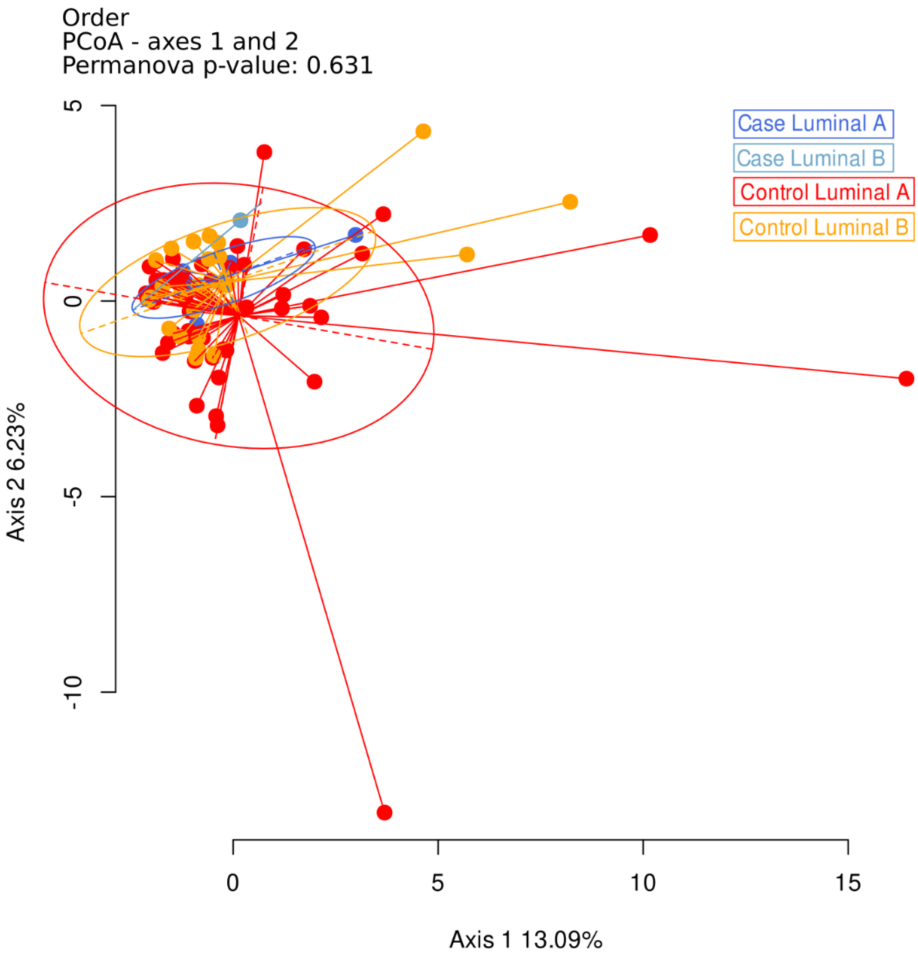
Order
PCoA - axes 1 and 2
Permanova p-value: 0.631
Case Luminal A
Case Luminal B
Control Luminal A
Control Luminal B
Axis 2 6.23%
5
0
-5
-10
0
5
10
15
Axis 1 13.09%

(b)

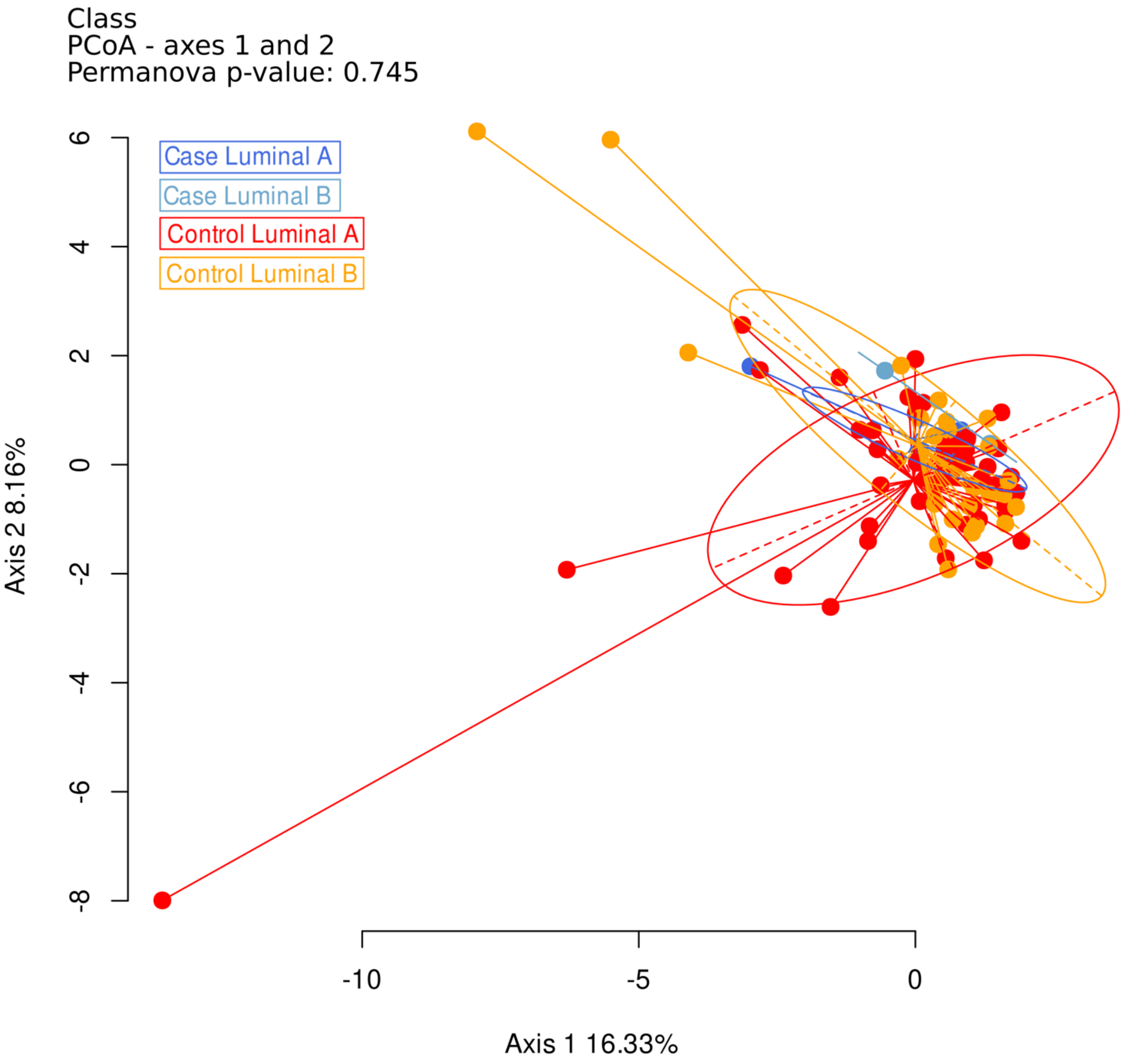

(c)

Supplementary Figure S1. Beta-diversity according to Luminal A and luminal B (case and control group). The microbiota distances were evaluated through the Bray–Curtis dissimilarity matrix at the taxonomic level of phylum (a), order (b) and class (c) and visualized through Principal Coordinates Analysis (PCoA). Each point represents the microbiota composition of one sample.
