# Supplementary material for: The Bio-Diversity and the Role of Gut Microbiota in Postmenopausal Women with Luminal Breast Cancer Treated with Aromatase Inhibitors: An Observational Cohort Study
Source: Pathogens. 2022 Nov 26;11(12):1421. doi: 10.3390/pathogens11121421 (PMC9781910; doi:10.3390/pathogens11121421)
Supplement: Supplementary file 1 [file pathogens-11-01421-s001.zip › Figure S2.pdf]

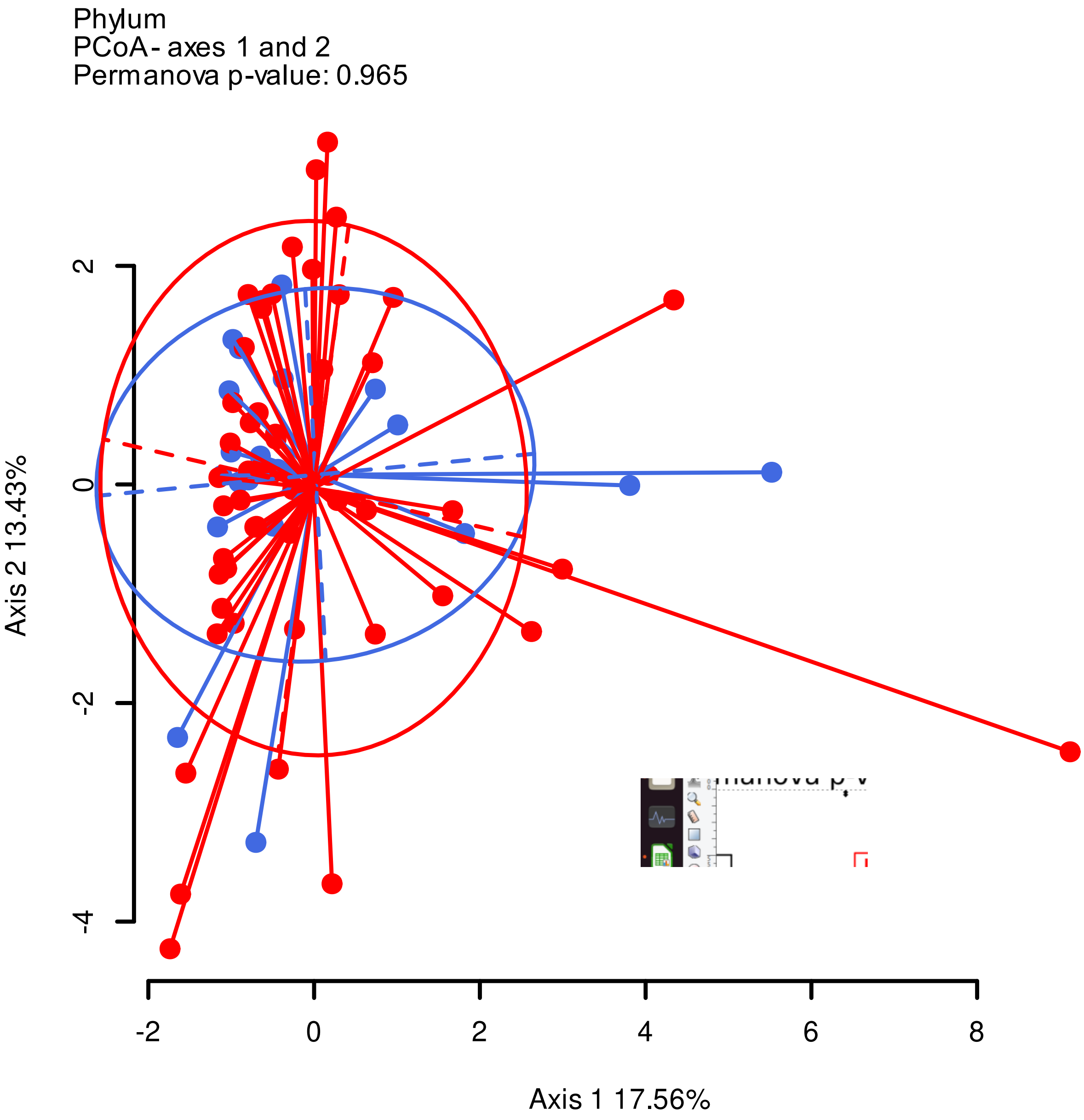
Phylum
PCoA- axes 1 and 2
Permanova p-value: 0.965
Axis 2 13.43%
2
0
-2
-4
-2
0
2
4
6
8
Axis 1 17.56%

(a)

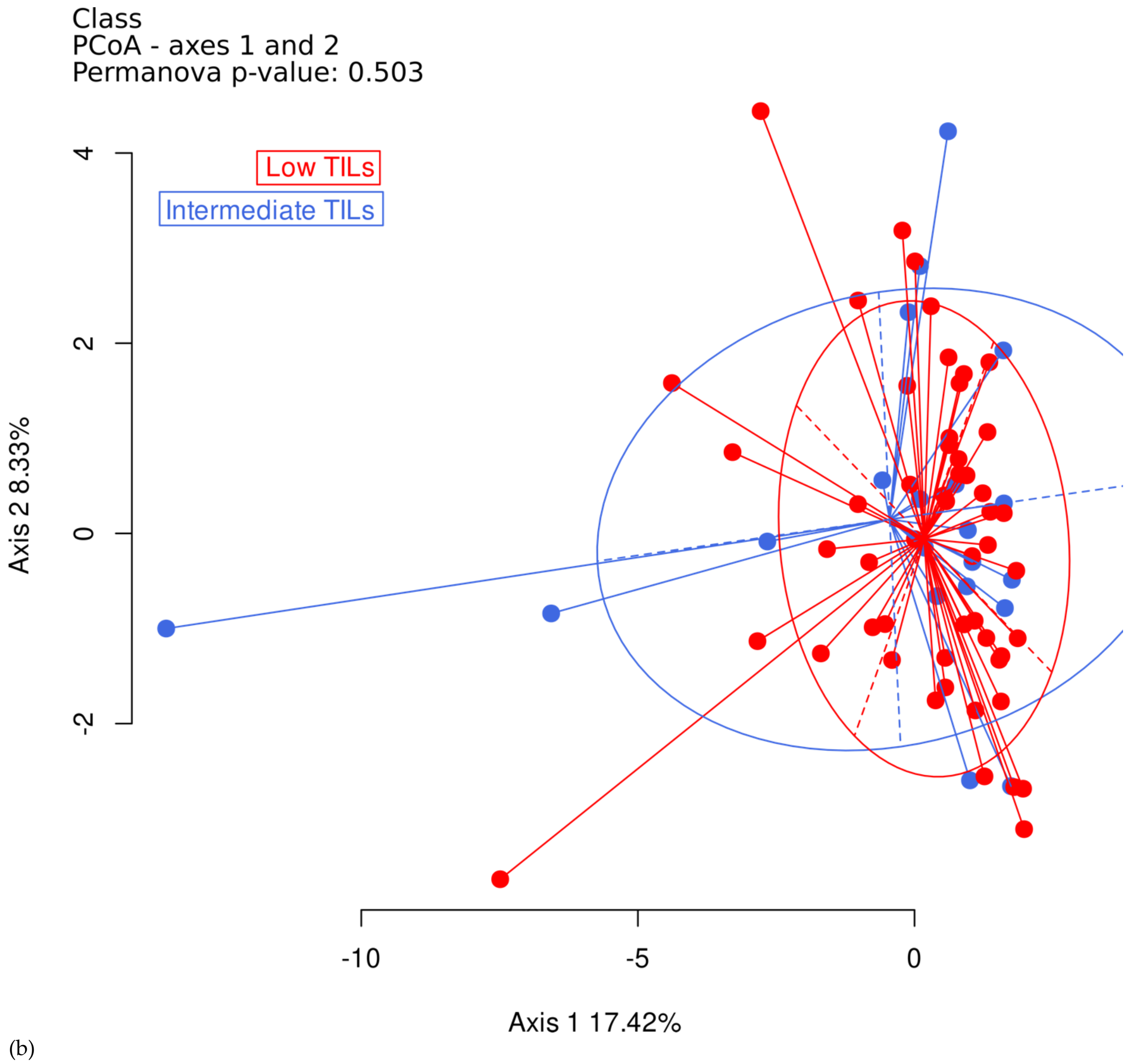

Class
PCoA - axes 1 and 2
Permanova p-value: 0.503
Low TILs
Intermediate TILs
Axis 2 8.33%
Axis 1 17.42%
4
2
0
-2
-10
-5
0

(b)

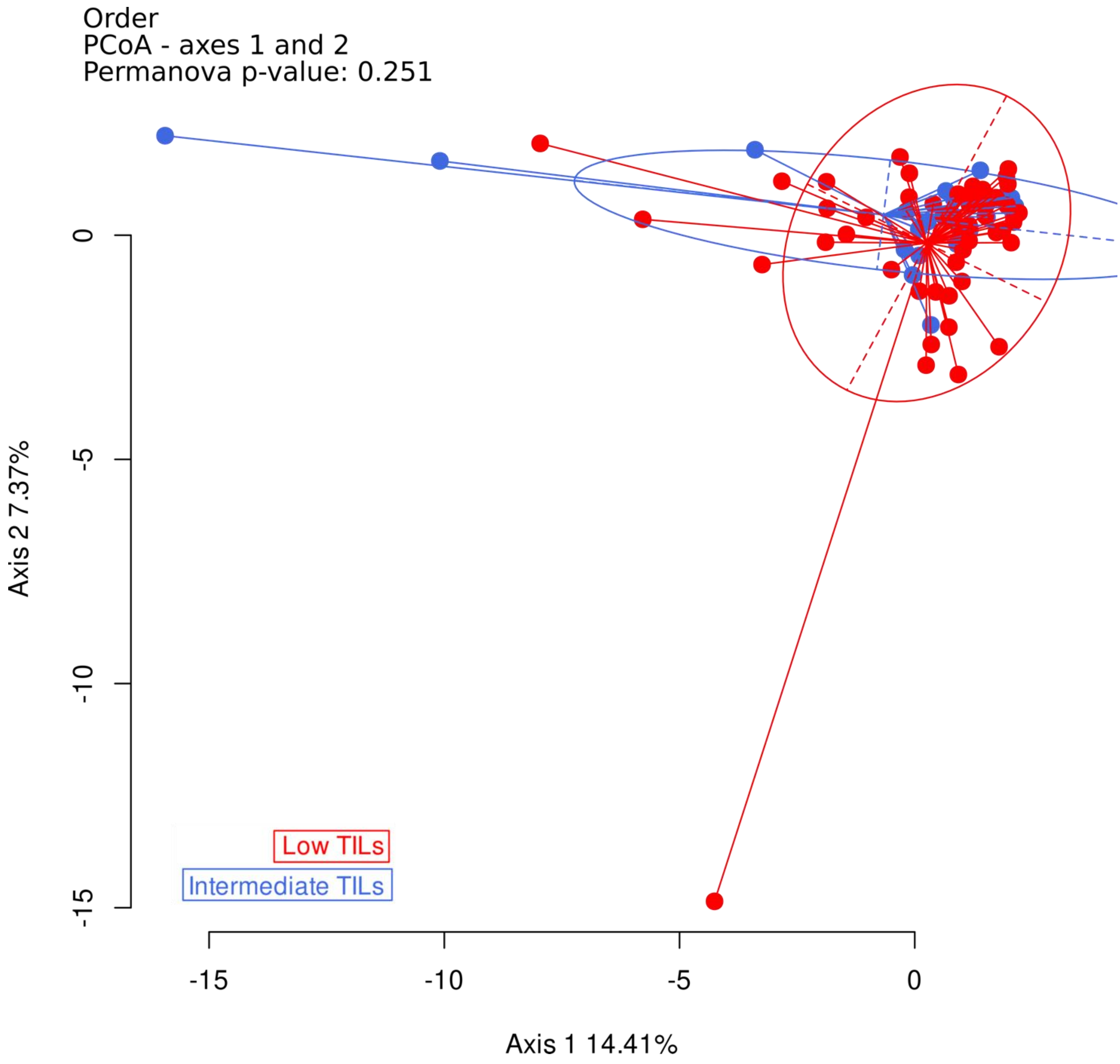

(c)

Supplementary Figure S2. Beta-diversity according to TILs levels. The microbiota distances were evaluated through the Bray–Curtis dissimilarity matrix at the taxonomic level of phylum (a), class (b) and order (c) and visualized through Principal Coordinates Analysis (PCoA). Each point represents the microbiota composition of one sample.
